# Influence of Health Literacy on the Physical Activity of Working Adults: A Cross-Sectional Analysis of the TRISEARCH Trial

**DOI:** 10.3390/ijerph16244948

**Published:** 2019-12-06

**Authors:** Kevin Rudolf, Bianca Biallas, Lea A. L. Dejonghe, Christopher Grieben, Lisa-Marie Rückel, Andrea Schaller, Gerrit Stassen, Holger Pfaff, Ingo Froböse

**Affiliations:** 1 Institute of Movement Therapy and Movement-Oriented Prevention and Rehabilitation, German Sport University Cologne, Am Sportpark Muengersdorf 6, 50933 Cologne, Germany; 2 Working Group Physical Activity-Related Prevention Research, German Sport University Cologne, Am Sportpark Muengersdorf 6, 50933 Cologne, Germany; 3 Institute for Medical Sociology, Health Services Research, and Rehabilitation Science (IMVR) of the University of Cologne, Eupener Straße 129, 50933 Cologne, Germany

**Keywords:** health literacy, physical activity, association, health behavior, accelerometry, questionnaire, work, adults, health promotion

## Abstract

Studies show that high health literacy (HL) can support the promotion and maintenance of healthy behavior such as physical activity (PA). However, most studies rely on subjective data. The aim of the present study is to investigate the relationship between HL and PA, not only with subjectively but also with objectively measured PA data. The present study is a pooled analysis of baseline data from the research association TRISEARCH (2015–2018), which focused on the HL of working adults. HL was measured by Lenartz’ questionnaire, and PA by the Global Physical Activity Questionnaire (GPAQ; n = 1056). A subsample (n = 124) also received accelerometers (Actigraph GT3X+) to provide more objective PA data. Partial correlations and regression models were used to investigate the relationship between HL and questionnaire- and accelerometer-derived PA. Very low and medium partial correlations could be found for HL subscales and daily PA by questionnaire (r = −0.06, *p* < 0.05) and accelerometer (r = 0.26, *p* < 0.01). No subscale of HL made a significant contribution to the amount of daily PA (all *p* > 0.05). Not all subscales of HL seem to have an influence on the occurrence of healthy behavior, such as PA. This should be considered when HL-based interventions are designed. Further investigation of the relationship between HL and PA is needed. Objective assessments of both HL and PA can provide additional information for this task.

## 1. Introduction

Health-related competencies or health literacy (HL) are defined as “cognitive and social skills which determine the motivation and ability of individuals to gain access to, understand and use information in ways which promote and maintain good health” [1]. Studies show that high HL is associated with healthier lifestyles and health-promoting and -maintaining behaviors in healthy adults [2] as well as in people with cardiovascular [3] or metabolic diseases [4]. In recent years, many studies have investigated the quality of HL in the population. The “European Health Literacy Survey” (HLS-EU) showed that almost one in two respondents (47%) have insufficient HL [5]. In regard to the different areas of HL, a study of the German population showed that 20.8% of the respondents had inadequate HL in the field of health-promoting behavior, compared to 11.5% for prevention and 10.3% for dealing with diseases [6]. 

**Figure 1:**
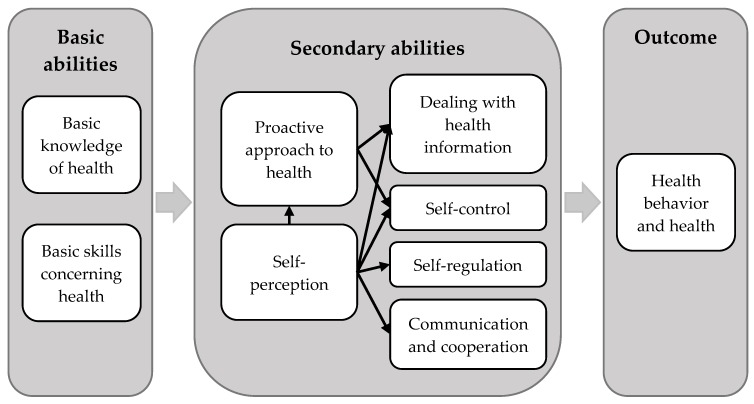
Structural model of health literacy (HL) according to Lenartz [7] and Soellner et al. [8].

The validated structural model of HL introduced by Lenartz [7] and Soellner et al. [8] (see Figure 1) provides a possible theoretical explanation of the influence of HL on health behavior. In short, *health behavior and health* are the result of two basic and six secondary abilities, all of which define HL. In this model, *basic knowledge of health* and *basic skills concerning health* build the foundation, e.g., the ability to read health-related manuals [9] and understand terms describing the body [7]. These basic abilities influence the secondary abilities. Health-related *self-perception* includes the perception of one’s own needs and feelings as well as the focus on internal and external processes (e.g., being aware of tensing up in certain situations), which again are part of *self-regulation* [8,10]. Together with the willingness and ability to take responsibility for one’s own health (*proactive approach to health*), *self-perception* influences even more secondary abilities: *Dealing with health information* describes the capability to understand health-related information and integrate it into one’s personal life. Someone with high *self-control* is disciplined in pursuing personal goals [11], whereas regulatory processes help to deal with self-congruent and -incongruent topics (*self-regulation,* e.g., being able to relax) [11]. Lastly, *communication and cooperation* describes behavior which intends to establish and maintain good relationships with others [7]. 

One of the most common methods in maintaining health and decreasing the risk of non-communicable diseases is physical activity (PA) [12,13,14,15,16,17]. According to Lenartz’ model, people who have higher HL should also show more health behavior than people who are less health literate. Since PA is acknowledged as a healthy behavior, it is questionable if physically active people show greater occurrences of HL and vice versa. In fact, some studies show that training in HL for healthy adults [2] as well as adults with chronic diseases [18] leads to increased PA levels afterwards, thus supporting this assumption. However, the majority of results on the association between HL and PA were obtained with questionnaires and overall HL scores [18]. Up to now, the association between objective PA data and HL has only been scarcely investigated in adults [19,20]. Therefore, the aim of the present study is to investigate the relationships between the different aspects of HL and PA, not only with subjectively (questionnaire) but also with objectively (accelerometry) measured PA data. In line with the literature [2,3,4,18] and the structural model by Lenartz [7], we hypothesize a positive correlation between HL and both measures of PA.

## 2. Materials and Methods 

### 2.1. Study Design

The present study is a pooled analysis of baseline data from studies by the research association TRISEARCH, which had the overarching objective to assess and improve HL in different target groups in the workplace [21]. For the present evaluation, data of working adults with health-related risk factors (AtRisk; [22]), apprentices in the vocational school setting (Web-App; [23]), and industry managers (HeLEvi; [10]) were used. Detailed eligibility criteria for the participation in the individual studies can be found in the respective publications [22,23,24]. Inclusion criteria for the present analysis were age between 18 and 65 years, inclusion in only one of the TRISEARCH studies and baseline assessments, and the completion of both questionnaires on HL and PA.

### 2.2. Measures

Data collection took place between 2015 and 2018 within the baseline assessments of the three studies. All participants provided written informed consent.

#### 2.2.1. Health Literacy

HL was derived from the German questionnaire by Lenartz [7]. The questionnaire is a self-report instrument which requires the participants to rate 29 health statements on a four-point Likert scale from 1 (strongly disagree) to 4 (strongly agree). In line with Lenartz’ model, the questionnaire offers a differentiation of six subscales: *self-regulation* (e.g., “I can easily switch between phases of high concentration and phases of relaxation”)*, self-control* (e.g., “When working on a task, I can prevent my thoughts from constantly wandering off”)*, self-perception* (e.g., “If I feel uncomfortable, I usually know exactly why”)*, proactive approach to health* (e.g., “I take good care of my body”)*, communication and cooperation* (e.g., “When I am not feeling well, I have no problem accepting someone’s help”)*,* and *dealing with health information* (e.g., “Information about health is often unclear to me”) [8]. The score of the HL subscales is calculated by generating the mean value of the subscales underlying items. The questionnaire was shown to be a reliable and valid instrument for the assessment of HL in different samples [7,25].

#### 2.2.2. Physical Activity

The subjectively perceived amount of PA was measured by the Global Physical Activity Questionnaire (GPAQ) [26]. The GPAQ is a valid measurement for assessing moderate to vigorous PA (MVPA) in a typical week (r = 0.20–0.48) [27,28,29] and therefore provides information about the duration (minutes) and intensity of PA. It has 16 items in total, which address moderate and vigorous PA at work (six items), moderate intensity travel to and from places (three items), moderate and vigorous recreational PA (six items) and sedentary behavior (one item). Scores (minutes per week or day) can be calculated, for example, for overall MVPA, MVPA in each domain, overall moderate intensity PA, overall vigorous intensity PA, and daily sedentary behavior.

A subsample of each subproject additionally received accelerometers (Actigraph GT3X+) to provide objective data on PA. Wearing the accelerometer was voluntary, as a previous study [30] showed no impact of this procedure on resulting data. The Actigraph GT3X+ measures movement among three axes which are summed up as so-called counts per minute (CPM). The device is reliable in the assessment of PA in adults [31] and is validated against heart rate telemetry (r = 0.66–0.82) [32], indirect calorimetry (r = 0.66–0.88) [32,33] and the doubly labeled water method (r = 0.26–0.58) [34]. The participants were instructed to wear the device on their right hip over a typical week during all activities except swimming, bathing or showering. The data were recorded with 60 Hz and saved in 30-second epochs. The data were used if the active wear time exceeded 10 hours on at least three days. Freedson [35] and Troiano [36] algorithms were applied allowing a classification of CPM into movement intensities. Zero to 99 CPM was classified as sedentary behavior, 100 to 1951 CPM as light intensity, 1952 to 5724 CPM as moderate intensity and everything above as vigorous intensity [35]. In this way, duration and intensity of the participants’ behavior was calculated. For the present analyses, however, moderate to vigorous PA (MVPA) was used if it exceeded ten minutes duration with a maximal interruption of two minutes [37].

On the basis of GPAQ and Actigraph data, daily and weekly minutes of MVPA as well as daily MET (metabolic equivalent of task) of MVPA were calculated. The MET refers to the energy cost of an activity [38], where a higher value symbolizes a more intense activity. For example, moderate intensity equals an average MET value of 4, which represents a four times higher activity compared to resting behavior. In the same way, vigorous intensity was calculated with a MET value of 8.

### 2.3. Statistical Analyses

Since no instruction for handling missing values was provided by Lenartz [7], the authors decided that missing values in the HL subscales would be imputed by using the mean of the subscale’s other items. However, only one missing value per subscale was allowed. Cases with more missing values were excluded from the analysis. The analysis of GPAQ was only done with complete data. Listwise deletion was used for all analyses. 

Descriptive statistics (frequencies, percentages, means and standard deviations) were calculated for demographic characteristics, HL and PA based on the questionnaires and accelerometers. For the investigation of the overall relationship between PA and HL, partial correlations were used for the objective and subjective measurement of PA and the subscales of the Lenartz questionnaire. Since previous studies have shown that physical activity and health literacy differ by sex and age [5,39,40], sex and age were entered as covariates. Correlation coefficients were interpreted as “low” (r = 0.10–0.19), “medium” (r = 0.20–0.29) and “large” (r ≥ 0.30) [41].

Regression models were used to investigate the influence of HL on the amount of PA. For this purpose, two linear regression models were used for subjectively and objectively assessed MVPA as dependent variables, respectively, and the subscales of HL (*self-regulation, self-control, self-perception, proactive approach to health, communication and cooperation* and *dealing with health information*), sex (male vs. female) and age as independent variables. The analyses were performed for both objective and subjective measures. 

The significance level was set at *p* < 0.05 for all tests, and the data were analyzed using IBM SPSS Statistics 25.

## 3. Results

### 3.1. Descriptive Statistics

**Figure 2:**
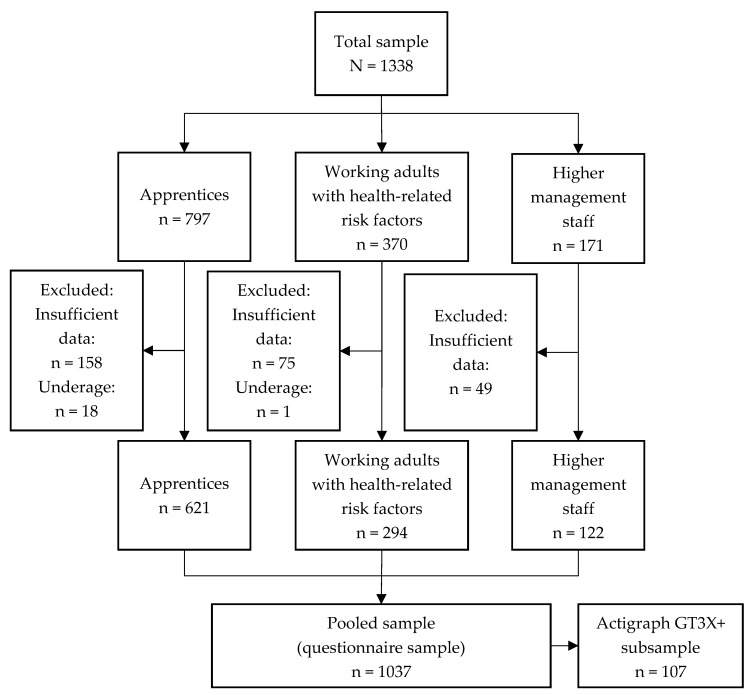
Flow chart of drop-out rates among the three study branches.

In all primary studies, a total of 1338 participants, 590 (44.1%) of them male, mean age 32.5 (14.5) years, were recruited. After applying the eligibility criteria for the present analyses, 1037 participants were included in the pooled sample. Of those, a total of 156 participants (15.0%) received an accelerometer and 107 provided sufficient data. Figure 2 shows the flow chart illustrating the participation and drop-out rate of the pooled sample. The demographic characteristics of each sample are displayed in Table 1.

Descriptive statistics for the subjective and objective PA measurements are described in Table 2. For the main outcome MVPA, participants reported 102.0 (141.6) minutes of MVPA per day. Of all self-reported activities, on average, 22.8% were work-related, 31.3% transport-related and 45.9% were leisure time activity. The subsample which was additionally wearing an Actigraph GT3X+ accumulated 59.4 (40.1) minutes of MVPA per day. The results of the HL questionnaire are reported in Table 3. For each of the subscales *self-regulation*, *proactive approach to health* and *communication and cooperation*, a mean value of 2.6 (0.6) was calculated. The mean value for the subscale *self-control* was 2.9 (0.5), for *self-perception* was 3.0 (0.5), and for *dealing with health information* was 3.0 (0.6).

### 3.2. Relationship between PA and HL

For the subjective measurement, the subscale *self-perception* showed a very low negative partial correlation with daily MET of MVPA (r = −0.06, *p* < 0.05) and daily vigorous PA (r = −0.08; *p* < 0.05).

When looking at the data drawn from the accelerometers, one statistically significant medium positive partial correlation between the subscale *proactive approach to health* and daily vigorous PA (r = 0.25, *p* < 0.01) was found. All other partial correlation analyses were non-significant (*p* > 0.05). 

### 3.3. Influencing Factors on the Amount of MVPA

For both regression analyses, no aspect of HL made a significant contribution to the amount of daily MVPA (all *p* > 0.05, GPAQ: adjusted R² < 0.01, Actigraph GT3X+: adjusted R² = 0.06, see Table 4 and Table 5). Only age made a significant positive contribution to the amount of objectively measured daily MVPA (Beta: 0.64; *p* < 0.05).

## 4. Discussion

The aim of the present study was to investigate whether a relationship between HL and PA measured by subjective and objective instruments exists. In total, the study population reported moderate to high values for HL and PA. The hypothesized positive correlation between HL and PA was only partly confirmed. Only one very low correlation could be observed for the subjective assessment of PA and one subscale of HL (*self-perception*). In regard to the objective PA levels, one medium correlation with the subscale *proactive approach to health* was found. Since the majority of HL subscales showed no connection to PA at all, these results indicate that not all aspects of HL might be equally important for promoting and maintaining healthy behavior.

The present study only partly confirms previous research which showed a connection between HL and PA [19,20,42]. Neither the regression analyses nor the majority of the correlations showed significant associations between the subscales of HL and objective and subjective PA. Only the *proactive approach to health* was statistically significantly correlated to objectively measured vigorous activity. A possible explanation for the correlation found might be that, according to Lenartz [7], the *proactive approach to health* includes having the awareness that health is not given but something that has to be worked for. One might assume that the *proactive approach to health* plays an important role in participating in more intense activities, e.g., swimming or jogging [43]. Such activities are mostly intentionally performed and planned. Therefore, it is likely that people taking responsibility for their own health engage in sport activities purposely. Previous studies on self-determination theory [44] have shown that engagement in PA behavior increases if a person has autonomous forms of motivation and actively takes responsibility for their health [45,46]. In line with this, a more detailed analysis of our results revealed that a *proactive approach to health* correlates with leisure time MET minutes (r = 0.31, *p* < 0.001) but not with work time MET minutes. This supports the assumption that if the importance of health-promoting behavior is internalized and a person takes active responsibility for showing such behavior, increased vigorous PA might be the result and be shown in leisure time.

In addition to those findings, a result that is controversial in relation to the literature was found: Lenartz’ assumptions and previous results, which postulated that a greater ability in perceiving inner and outer processes results in a healthier lifestyle and adaptive health behavior [47], could not be affirmed. While the subjective PA data revealed only very low negative correlations with *self-perception,* neither the regression analyses nor the data from the accelerometry showed any connection at all. A possible explanation for these results may refer to the perception of PA. As previous studies have shown, self-reported PA in terms of intensity and duration differs from data recorded by more objective tools [48,49]. PA of moderate or vigorous intensity is often overestimated while sedentary behavior is frequently underestimated in relation to the respective objective data [50]. Therefore, it stands to reason whether people with high self-perception are more precise when reporting their own PA. In this way, their reported PA might be lower than that of people with worse self-perception. This assumption is supported by psychology research which concludes that respondents are more accurate in their ratings when they are more self-aware [51,52] as well as by the fact that no influence of *self-perception* on the data measured by accelerometry was found. 

Although the strongest correlation between HL and PA was found within the Actigraph GT3X+ data, it is questionable whether the objective measurement and the resulting more valid estimation of PA [53] has a connection to general HL. Only a few HL studies have assessed PA with an objective device so far [2,19,20]. Al Sayah et al. [19] found a significant interaction between HL and subjectively assessed PA, but no interaction between HL and objectively measured PA. A study by Aldana et al. [2] was able to observe a significant increase in PA by 30% after a health educational course. However, no correlation analysis between HL and PA was provided. Riecken [20], on the other hand, was able to observe a significant correlation between HL and objectively assessed PA. Hence, the question of whether all or only certain aspects of HL relate to objective PA and PA in general is still unanswered. Further research with objective measures and larger sample sizes is needed to establish solid evidence regarding the relationship between HL and PA behavior. 

### Limitations

The present study was conducted as a pooled analysis of three projects by the research association TRISEARCH, which were addressing different target groups. Due to the cross-sectional study design, testing the direction of causality between HL and PA was not possible. Moreover, large differences between the different target groups regarding age and sex were present. One project was conducted with apprentices, who were at the start of their working career and, therefore, much younger. Research showed that younger age was a significant predictor for lower HL levels [54]. Additionally, the target group of working adults with health-related risk factors participated in a rehabilitation and prevention program, which was able to offer external support. Even if our population showed great heterogeneity, age and sex were entered as covariates into the analyses and therefore controlled. However, since not all studies recorded data on participants’ educational levels, body-mass index or socio-economic status, we could not include these as possible covariates. Hence, a potential bias cannot be ruled out.

In the present study, HL was assessed with a questionnaire generated by the German researcher Lenartz. This questionnaire is not yet as commonly used as the Health Literacy Questionnaire (HLQ) [55]. The HLQ is a valid and reliable measurement of HL [55,56,57]. In comparison to the HLQ, the Lenartz questionnaire fails in providing a global HL score but offers a differentiation on six subscales compared to the nine of the HLQ. This makes the questionnaire more compact and feasible. Furthermore, the Lenartz questionnaire focuses on the respondent’s self, whereas the HLQ focuses on dealing with the outside world and being resourceful [55]. Additionally, the connection to the HL model of Lenartz [7], which was used as a theoretical basis in the TRISEARCH projects, led to the decision to use the Lenartz questionnaire. 

A problem that might occur when both PA and HL are measured via questionnaires is that both measurements underlie the probability of recall and social desirability bias. Recall or social desirability bias could, for instance, influence the participants’ response when recalling the length and occurrence of PA behavior [53]. People tend to over-report their PA behavior [50,58] and might be likely to show the same pattern in HL questionnaires. This could lead to a higher probability of gaining significant correlations between these two instruments. Our study is one of the few existing studies which included objective and subjective PA measurements, although, only a subsample had access to accelerometry because of limited resources and compliance of participants. The compliance of the participants wearing the accelerometer was also the reason why we chose the inclusion criterion of at least three days with at least ten hours of recorded data. The widely used recommendation by Trost et al. [59] of at least 4 valid days, including at least one weekend day, would have further reduced the sample size to 92 participants. Nevertheless, the results regarding the association between HL and PA, as well as the influence of the HL subscales on the amount of MVPA, would have been similar. Thus, the inclusion criterion of three days was kept to maintain the larger sample size.

Due to these limitations, the generalizability of the Actigraph GT3X+ results must be treated with caution. Hence, further studies with objective measures of HL and healthy behavior are needed to improve the understanding of the underlying mechanisms of this relationship.

## 5. Conclusions

The present study suggests that not all subscales of HL influence the occurrence of healthy behavior—such as PA—in the same way. More research on the different components of HL and their particular influence on specific health behaviors is needed to improve the quality of HL-based interventions for promoting healthy behavior. As the results indicate, making sure that people take responsibility for their own health seems like a promising approach. 

## Figures and Tables

**Table 1:** Demographic characteristics for the different samples.

	**Demographic Characteristics of the Total Sample**			
	All (n = 1338)	Apprentices (n = 797)	Working adults with health-related risk factors (n = 370)	Industry managers (n = 171)
Sex [male] n (%)	590 (44.1%)	292 (39.0%)	181 (49.1%)	117 (80.7%)
Age [years] mean (SD)	32.5 (14.5)	21.8 (4.7)	47.4 (10.0)	49.3 (6.0)
	**Demographic Characteristics of the Pooled Sample**			
	All (n = 1037)	Apprentices (n = 621)	Working adults with health-related risk factors (n = 294)	Industry managers (n = 122)
Sex [male] n (%)	485 (46.8%)	245 (39.5%)	142 (48.3%)	97 (79.5%)
Age [years] mean (SD)	32.3 (14.3)	22.0 (4.7)	47.0 (9.9)	49.3 (5.9)
	**Demographic Characteristics of the Actigraph GT3X+ Subsample**			
	All (n = 107)	Apprentices (n = 32)	Working adults with health-related risk factors (n = 45)	Industry managers (n = 30)
Sex [male] n (%)	46 (43.0%)	7 (21.9%)	17 (37.8%)	22 (73.3%)
Age [years] mean (SD)	41.1 (14.9)	21.2 (4.3)	48.5 (9.4)	51.3 (4.7)

Percentages are valid percentages.

**Table 2:** Data of the PA measurements.

	**Subjective Measurement (Global Physical Activity Questionnaire, GPAQ)**			
	**All (n = 1037)**	**Apprentices (n = 621)**	**Working Adults with Health-Related Risk Factors (n = 294)**	**Industry Managers (n = 122)**
Daily moderate to vigorous physical activity (MVPA) [min] mean (SD)	102.0 (141.6)	97.5 (119.8)	136.9 (192.7)	41.0 (36.7)
Daily metabolic equivalent of task (MET) of MVPA [min] mean (SD)	551.8 (777.8)	543.1 (665.5)	701.7 (1057.3)	234.2 (208.6)
Daily vigorous physical activity (PA) [min] mean (SD)	35.9 (66.1)	38.3 (58.9)	38.6 (88.3)	17.5 (19.0)
Daily moderate PA [min] mean (SD)	66.1 (105.8)	59.2 (89.1)	98.3 (141.4)	23.5 (26.4)
	**Objective Measurement (Actigraph GT3X+)**			
	**All (n = 107)**	**Apprentices (n = 32)**	**Working Adults with Health-Related Risk Factors (n = 45)**	**Industry Managers (n = 30)**
Daily MVPA [min] mean (SD)	59.4 (40.1)	41.4 (13.6)	66.6 (48.1)	67.9 (40.7)
Daily MET of MVPA [min] mean (SD)	253.7 (174.2)	176.8 (63.9)	281.0 (207.4)	294.7 (178.6)
Daily vigorous PA [min] mean (SD)	4.0 (5.6)	2.8 (3.9)	3.7 (6.0)	5.7 (6.4)
Daily moderate PA [min] mean (SD)	55.4 (37.2)	38.6 (11.9)	62.9 (44.8)	62.2 (37.5)

Percentages are valid percentages.

**Table 3:** Descriptive statistics of the HL questionnaire.

	Health Literacy (Lenartz’ Questionnaire) [Scale 1–4]			
	All (n = 1037)	Apprentices (n = 621)	Working Adults with Health-Related Risk Factors (n = 294)	Industry Managers (n = 122)
Self-perception mean (SD)	3.0 (0.5)	3.0 (0.5)	2.9 (0.6)	3.0 (0.5)
Proactive approach to health mean (SD)	2.6 (0.6)	2.6 (0.6)	2.6 (0.6)	2.9 (0.5)
Dealing with health information mean (SD)	3.0 (0.6)	2.9 (0.6)	3.0 (0.6)	3.2 (0.6)
Self-control mean (SD)	2.9 (0.5)	2.9 (0.5)	2.8 (0.6)	3.1 (0.4)
Self-regulation mean (SD)	2.6 (0.6)	2.7 (0.6)	2.5 (0.6)	2.5 (0.6)
Communication and cooperation mean (SD)	2.6 (0.6)	2.6 (0.6)	2.6 (0.7)	2.7 (0.6)

**Table 4:** Regression analysis of influencing factors on subjective MVPA (GPAQ).

n = 1037	Beta	SE (β)	T	Sig.	95%-CI
**Self-perception**	−4.32	10.22	−0.42	0.67	[−24.36–15.73]
**Proactive approach to health**	14.35	8.09	1.77	0.08	[−1.54–30.23]
**Dealing with health information**	−15.22	8.40	−1.81	0.07	[−31.70–1.27]
**Self-control**	0.84	9.77	0.09	0.93	[−18.32–20.01]
**Self-regulation**	−11.70	8.48	−1.38	0.17	[−28.33–4.93]
**Communication and cooperation**	−4.32	7.48	−0.58	0.56	[−19.00–10.35]
**Sex (male vs. female)**	10.50	9.29	1.13	0.26	[−7.73–28.73]
**Age**	0.23	0.33	0.70	0.48	[−0.41–0.87]

Dependent variable: subjective daily MVPA, adjusted R² < 0.01.

**Table 5:** Regression analysis of influencing factors on objective MVPA (Actigraph GT3X+).

n = 107	Beta	SE (β)	T	Sig.	95%-CI
**Self-perception**	−9.37	9.41	−1.00	0.32	[−28.04–9.31]
**Proactive approach to health**	14.08	9.06	1.55	0.12	[−3.89–32.05]
**Dealing with health information**	0.13	9.04	0.01	0.99	[−17.81–18.06]
**Self-control**	−9.74	9.57	−1.02	0.31	[−28.74–9.26]
**Self-regulation**	−6.08	7.51	−0.81	0.42	[−20.98–8.83]
**Communication and cooperation**	−0.75	6.29	−0.12	0.90	[−13.24–11.73]
**Sex (male vs. female)**	8.23	8.57	0.96	0.34	[−8.78–25.24]
**Age**	0.64	0.30	2.14	0.04	[0.05–1.23]

Dependent variable: objective daily MVPA, adjusted R² = 0.06.
